# Minoritized students and their faculty research mentors view benevolence differently in the relationship

**DOI:** 10.1371/journal.pone.0332153

**Published:** 2025-09-09

**Authors:** Star W. Lee, Haley Miyasato, Jocelyn Tirado, Stephanie Dingwall, Richard A. Cardullo

**Affiliations:** 1 Department of Developmental and Cell Biology, University of California, Irvine, California, United States of America; 2 Department of Biochemistry, University of California, Riverside, California, United States of America; 3 Department of Evolution, Ecology, and Organismal Biology, University of California, Riverside, California, United States of America; Institute of Medical Biochemistry Leopoldo de Meis (IBqM) - Federal University of Rio de Janeiro (UFRJ), BRAZIL

## Abstract

There are many benefits for students who participate in undergraduate research experiences, including increased retention and persistence in science, technology, engineering, and mathematics (STEM). By doing research, minoritized students increase their likelihood of pursuing graduate school and STEM careers. The benefits of research experiences are partially mediated by students’ interactions with their faculty research mentor. Building trust in the relationship requires students to believe that their faculty mentors are both competent and caring. Here, we used a mixed-methods approach to evaluate the relationship between students and their research mentors. We surveyed both minoritized students’ and their faculty mentors’ perceptions of the mentor’s ability and benevolence. Students rated the faculty mentors’ abilities higher than how mentors rated themselves. In contrast, students rated the faculty mentors’ benevolence significantly lower than how mentors rated themselves. In follow-up interviews focused on benevolence, students emphasized that faculty mentors demonstrated caring through instrumental support (i.e., research skills or career guidance); faculty mentors described providing psychosocial (i.e., social or emotional) support to students. Our results show that there was a difference in how minoritized students and their faculty mentors communicate care in mentor-mentee relationships in research. Findings from this study indicate how faculty mentors may better support minoritized students in undergraduate research experiences.

## Introduction

In 2025, there has been an abrupt reversal in how the federal government of the United States supports the diversification of different fields. The recent actions against diversification will stifle innovation and creativity, which are strongly contributed by people with different backgrounds and experiences [1,2]. Historically, in science, technology, engineering, and mathematics (STEM), there have been multiple national calls for increased representation of people from various backgrounds [3–5]. The current STEM workforce does not reflect the nation’s diverse population of women, minorities, and people with disabilities [2]. While women and minoritized students are equally likely to start off as STEM majors, they are less likely to graduate with STEM degrees than their represented counterparts [6–8]. Persistence in STEM remains challenging for minoritized students who feel unwelcome, experience implicit and explicit bias, and encounter unsupportive environments [4,6,9–11].

Undergraduate research experiences (URE) represent a seminal experience where there are ample opportunities to support students and improve persistence in STEM. The benefits to participating in URE are multifaceted for students [5]. Students report gains in research skills (i.e., data analysis, lab techniques, etc.), self-confidence, and a sense of belonging [12]. Participating in URE increases the likelihood of retention and persistence in biology degrees for all students, and particularly impactful for minoritized students [13]. Minoritized students who participate in URE are more likely to graduate with a STEM degree, pursue graduate school, and have STEM careers compared to students without URE [14–16].

Students’ benefits from UREs depend on how they are mentored, and a major factor is whether students interact directly with their faculty research mentors [17]. Mentorship is an interpersonal relationship that requires trust between participants [18]. An effective mentor also provides both instrumental (career) and psychosocial support to their students [19,20]. Faculty mentors’ guidance is important in steering the careers of students, particularly minoritized students [21]. Minoritized students who interacted with faculty mentors frequently were more likely to have increased science identity (i.e., seeing oneself as a scientist) and intentions to pursue a STEM PhD compared to those students with minimal or non-existent faculty mentorship [22]. To increase the representation in STEM careers, improvement of mentoring for minoritized students in STEM research labs is needed [23]. Quality mentorship is positively associated with science identity, research skills, career knowledge, and science self-efficacy (i.e., beliefs in one’s ability to do science activities) for minoritized students [18,24–26].

Faculty play a critical role in providing students with both academic and interpersonal validation (demonstrating care and empathy) [27]. A positive interpersonal relationship between faculty and students is based on many factors, including credibility, caring, rapport, among others [28]. It is important for faculty mentors to support their students socioemotional health in STEM to build a more personal relationship [18,19,29]. Having a personal relationship with faculty mentors increases students’ sense of belonging in the lab [21]. Faculty mentors may support students psychosocially by valuing their students’ ideas and thinking [21]. Minoritized students feel closer to faculty mentors who are interested in their backgrounds and share personal information about themselves [30]. Without psychosocial support, students feel like their faculty mentors do not care about them [29].

Psychosocial support is an integral part of the mentoring relationship and helps to build trust between students and their mentors [18,31,32]. Trust is foundational to a positive relationship between faculty mentor and student, which takes time to develop [33]. Multiple factors, including ability and benevolence, contribute to building trust in a relationship [34]. In the context of mentoring, ability-based trust refers to beliefs that mentors are competent in their skills; benevolence-based trust refers to beliefs that mentors care about their mentee’s well-being [35]. A strong relationship is mediated by trust in both ability and benevolence [35].

Students’ trust in their faculty mentors is associated with positive student outcomes. Most research in this field has focused on the relationship between faculty mentors and graduate students. When minoritized graduate students feel trusted by their mentors, they feel more comfortable and more confident in their research [32]. A faculty mentor may increase students’ trust by being open and sharing personal experiences with their students [36]. Trust in the mentor is associated with increased minoritized students’ sense of belonging [37]. For minoritized undergraduate students, trust in their faculty mentors is positively associated with increased motivation and higher career expectations [38].

While there are several benefits to trusting faculty mentors, more research is needed to better characterize trust between minoritized undergraduate students and their mentors. In this study, we aimed to better understand how faculty mentors’ trustworthiness may be perceived by both minoritized students and faculty mentors themselves. We addressed the research questions listed below with a mixed-methods approach, using both quantitative and qualitative methods. We surveyed students in the University of California Louis Stokes Alliance for Minority Participation (CAMP) Scholars Program to determine if there are differences in the mentor’s ability and benevolence rated by minoritized students and their faculty mentors. We conducted follow-up interviews with a subset of participants to better understand their survey responses and further explore how benevolence is communicated between faculty mentors and students.

1. How do faculty mentors and minoritized students in STEM perceive the mentor’s ability and benevolence?
2. How do faculty mentors communicate benevolence to their minoritized students?

## Methods

### The University of California Louis Stokes Alliance for Minority Participation (CAMP) Scholars Program

The purpose of the CAMP program, supported through the National Science Foundation (NSF)-funded Louis Stokes Alliance for Minority Participation in STEM program, is to support and increase the number of minoritized STEM graduate students and professionals. The term “minoritized” is used intentionally to emphasize the ongoing systemic barriers faced by certain underrepresented groups. In this project, minoritized students include those from American Indian/Alaskan Native, Black/African-American, Native Hawaiian/other Pacific Islander, Hispanic/Latinx ethnic/racial groups. CAMP is an active program at all nine undergraduate University of California (UC) campuses. The CAMP program supports students in a multitude of ways, including research opportunities, financial support, academic advising, career counseling, professional development workshops, graduate school admission and preparation, and social activities. While the specific nature of CAMP programming differs among the UC campuses, all CAMP students must conduct faculty-mentored research at their respective campuses. In addition to learning laboratory skills, CAMP students develop and showcase their oral presentation skills at the annual UC-wide CAMP symposium, where they present talks or posters to their peers and STEM faculty from across the UC alliance.

### Study participants

The study was conducted in 2020–2023 and based at a large public university with a Carnegie basic classification of Doctoral University: Highest Research Activity. The study was conducted under the guidelines of the UC Riverside Institutional Review Board (protocol number 20–163). Recruitment for participants started February 25, 2021 and ended May 2, 2023. Informed consent was collected at the start of the survey (electronic) and interview (oral).

To be included in the study, student participants were enrolled at one of the nine undergraduate UC campuses (Berkeley, Davis, Irvine, Los Angeles, Merced, Riverside, San Diego, Santa Barbara, Santa Cruz) and were actively involved in faculty-mentored research in the CAMP program. Faculty research mentors were employed at one of the nine undergraduate UC campuses and mentored a student in the CAMP program. Study participants were required to speak English and be over the age of 18. Participants were not excluded based on any other demographic characteristics.

### Data collection

#### Survey development.

To determine whether there was a difference in how students and faculty mentors viewed faculty mentors, a survey was designed to focus on two factors: ability and benevolence of the mentor. The items were developed based on a review of the literature on ability and benevolence with respect to trust. Existing items were adapted from published surveys and modified for our CAMP students and faculty mentors. The measure of ability was focused on evaluating the mentors’ knowledge and skills in guiding students. For students, 3 items on ability included statements such as “This person is a knowledgeable teacher” and “This person is a good mentor”. Ability items were adapted from a published survey evaluating mentoring, including competence of mentors [38]. The 3 ability items were adapted to evaluate teaching, research, and mentoring skills.

The measure of benevolence was focused on evaluating how mentors’ care for students by taking a personal interest in them as individuals and being empathetic. Benevolence items were adapted from a published survey evaluating students’ perceptions of caring by a math instructor [39]. For students, 3 items on benevolence were adapted to be generic (“This person”) and included statements such as “This person cares about how I feel” and “This person takes a personal interest in me”. The 3 faculty items were similar but modified to “I care about how my student feels” and “I take a personal interest in my student”. Participants were asked to rate all items on a five-point Likert scale from 1 (strongly disagree) to 5 (strongly agree). Survey items for students and faculty are provided in S1 File.

#### Survey participants.

To determine participants’ perceptions of the faculty mentor’s ability and benevolence, CAMP students and their faculty mentors were provided with items on ability and benevolence in a survey. Surveys were administered to students (n = 111) and faculty (n = 86) once during the academic year following the annual UC-wide CAMP symposium. Demographic information for survey participants is provided in Table 1. Compared to faculty, student participants were more likely to identify as Black, t(171.06) = −2.22, p < 0.05, Latinx, t(180.09) = −10.05, p < 0.001, multi-racial, t(146.22) = −4.62, p < 0.001, or first-generation, t(158.96) = −3.58, p < 0.001. First-generation students are defined as students where neither parent nor guardian completed a baccalaureate degree [40]. Compared to students, faculty participants were more likely to identify as Asian, t(78) = 4.45, p < 0.001, or White, t(78) = 11.60, p < 0.001, compared to student participants.

**Table 1:** Demographic information for survey participants.

Category and Characteristic	Student % (n)	Faculty % (n)
Gender		
Female	65.8% (73)	41.9% (36)
Male	31.5% (35)	53.5% (46)
Non-binary	0.9% (1)	0.0% (0)
Decline to state	1.8% (2)	4.7% (4)
Race/ethnicity		
American Indian or Alaskan Native	0.9% (1)	1.2% (1)
Asian	0.0% (0)	18.6% (16)***
Black or African American	10.1% (11)*	2.3% (2)
Native Hawaiian or other Pacific Islander	0.9% (1)	1.2% (1)
Latinx	65.1% (71)***	8.1% (7)
White	0.0% (0)	58.1% (50)***
Multi-racial	22.9% (25)***	2.3% (2)
Decline to state	1.8% (2)	8.1% (7)
First-generation	69.4% (77)***	32.6% (28)
Transfer	31.5% (35)	9.2% (8)

*p < 0.05, ***p < 0.001

#### Interviews.

Based on the difference in how students and faculty mentors rated mentors’ benevolence in the survey, we were interested in learning more about how both groups viewed benevolence in the relationship and used interviews to validate survey responses and provide additional insight and explanations into participants’ responses. The use of multiple data sources guards against the study’s findings being an artifact of a single method or participants’ bias [41]. In the survey, students (n = 11) and faculty mentors (n = 10) indicated they were interested in doing a follow up interview. The interview occurred within 2–3 months of participants completing the survey. Demographic information for interview participants is provided in Table 2. Due to self-selection, only the student or faculty mentor in any relationship was interviewed. For example, a student may have participated in the interview, but their faculty mentor did not choose to participate in the interview. Interviews were semi-structured and participants were asked questions about the faculty mentor’s benevolence. For example, students were asked “Does your faculty research mentor care about how you feel? How do you know or how do they show you?”. Faculty were asked similar questions like “Do you care about how your student feels? How do they know you do or how do you show them?”. Interviews were conducted on Zoom and audio recorded. Recordings were transcribed and de-identified transcriptions were analyzed.

**Table 2:** Demographic information for interview participants.

Category and Characteristic	Student n	Faculty n
Gender		
Female	6	7
Male	5	3
Race/ethnicity		
Black or African American	1	0
Latinx	6	1
White	0	9
Multi-racial	4	0
First-generation	8	0
Transfer	2	2

Pseudonyms and demographic information for interview participants are provided in S1 Table. In addition to the difference in race/ethnicity backgrounds, 72.7% of students (n = 8) identified as first-generation students compared to 0.0% of faculty mentors (n = 0).

### Data analysis

#### Survey analysis.

The 3 items on ability had acceptable internal consistency (Cronbach’s alpha = 0.701) and 3 items on benevolence had good internal consistency (Cronbach’s alpha = 0.805) for this sample of 111 students and 86 faculty. The total for the 3 items on ability and 3 items on benevolence were determined from the surveys. The 3 item total score ranged from 0–15, with 15 being the highest. Welch’s *t* test was used to determine significant differences between students and faculty. Two-way ANOVA was used to evaluate interactions between gender and role or time spent in lab and role. All statistical analyses were performed using R.

#### Interview analysis.

The research team (H. M., J. T., and S. W. L.) took a thematic analysis approach to the interview data [42]. Once the interviews were transcribed, the research team independently read through three interviews from students and three interviews from faculty participants to identify possible themes. An inductive approach was used initially to generate a list of codes independently. Subsequently, initial codes were compared and discussed among the research team. After the team read through all the interviews, the team met repeatedly to discuss and categorize codes into different themes. Based on a process-oriented model of mentorship [18,19], deductive coding was used to differentiate between two major themes: instrumental support and psychosocial support of mentors. Inductive coding was used to identify additional themes. To develop the codebook, each code was defined together as a group. The researchers (H. M., J. T., and S. W. L.) met regularly to compare and revise codes until finalizing a codebook. Once the final codebook was developed, two researchers (H. M. and J. T.) coded the interviews using an iterative approach. They coded a third of the interviews independently and then met to discuss their analysis to reach a consensus for each interview. This process was repeated until all the interviews were analyzed accordingly.

The final codebook (S2 Table) consisted of two main themes: instrumental support and psychosocial support. Instrumental support codes focused on concrete ways faculty mentors supported students’ research and career aspirations. Main instrumental support codes included “access to resources”, “research collaboration”, and “goal setting & career planning”. Psychosocial support codes focused on methods used by faculty to support their students’ socioemotional health. Main psychosocial support codes included “rapport”, “community”, “addressing diversity”, and “flexibility”. Outside of these two main support themes, “barriers” was used to characterize experiences by students and faculty who identified a barrier or boundary between the two.

## Results

### Rating mentors’ ability and benevolence

**Fig 1:**
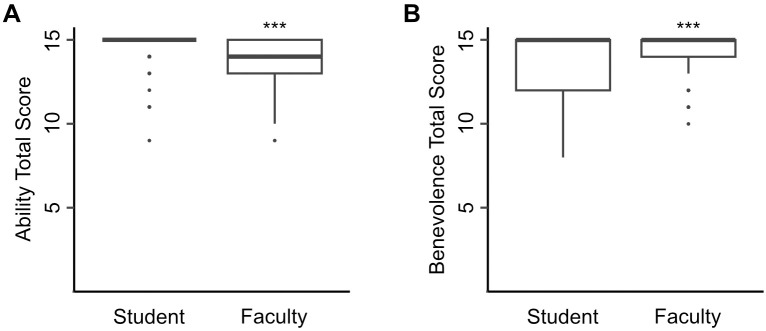
Comparison of total score for mentor’s ability and benevolence by students and faculty mentors. (A) Total score for mentor’s ability was rated higher by students (n = 111) than mentors (n = 86) (B) Total score for mentor’s benevolence was rated lower by students than mentors. The solid black line represents the median. *** p < 0.001.

Surveys were used to determine if there were differences in how students and faculty mentors perceive mentors’ ability and benevolence. Students rated their mentors’ ability highly, with a mean total score of 14.58 (SD 1.04, n = 111, Fig 1A) out of a maximum total of 15. Their rating of their mentors was significantly higher compared to the faculty mentor’s rating their own ability (mean 13.91, SD 1.29, n = 86) [t(160.81) = −3.93, p < 0.001]. Additional analysis revealed gender was not a factor in the ratings. There was no significant interaction between gender and role (student/faculty) on ratings of ability of mentors (2-way ANOVA, *F*(2, 190) = 0.55, p = 0.58). There was no statistically significant effect in ability by gender, *F*(3, 190) = 1.53, p = 0.21. There was a statistically significant effect in ability by role, *F*(1, 190) = 13.27, p < 0.001.

Students rated their mentors’ benevolence positively, with a mean total score of 13.40 (SD 2.17, Fig 1B). Their score for benevolence was significantly lower compared to faculty’s rating of their own benevolence (mean 14.24, SD 1.19) [t(177.16) = 3.49, p < 0.001] (Fig 1B). There was no significant interaction between gender and role on rating benevolence of mentors, (2-way ANOVA, *F*(2, 190) = 1.68, p = 0.19). There was no statistically significant effect in benevolence by gender, *F*(3, 190) = 0.61, p = 0.61. There was a statistically significant effect in benevolence by role, *F*(1, 190) = 9.92, p < 0.01.

Building a relationship takes time. To determine whether time was a factor in their rating for ability and benevolence, students and faculty were asked how long the students had been doing research in the lab. Of the 108 students surveyed, 32 (30%) reported being in the lab less than a year, 41 (38%) reported being in the lab 1–2 years, and 35 (32%) reported being in the lab more than 2 years. Of the 79 faculty surveyed, 14 (18%) reported their students being in the lab less than a year, 41 (52%) reported their students being in the lab 1–2 years, and 24 (30%) reported their students being in the lab more than 2 years. There was no difference in the amount of time reported by the students and faculty [t(179.6) = 0.91, p = 0.36]. Lastly, there was no significant interaction between time and role on the rating of mentors’ ability (2-way ANOVA, *F*(2, 181) = 0.68, p = 0.51) or benevolence (*F*(2, 181) = 0.39, p = 0.68). Time did not significantly impact their rating of mentor’s ability (*F*(2, 181) = 2.19, p = 0.12) or benevolence (*F*(2, 181) = 0.06, p = 0.94).

### Students’ perceptions of mentors’ benevolence

To better understand the difference in benevolence rating by students and faculty mentors in the survey, follow-up interviews were conducted and focused on how mentors communicated benevolence to students. We asked 11 students to describe how their mentors demonstrated caring to them. Students discussed positive experiences of instrumental and psychosocial support and negative experiences of barriers or feeling disconnected from their mentors.

#### Instrumental support.

Most students (n = 8) emphasized their mentors’ instrumental support, which focused on supporting their career or research skills. Students characterized instrumental support from the mentors through one of two main methods: access to resources and research collaboration (Table 3).

**Table 3:** Main subthemes of instrumental and psychosocial support emphasized by students and faculty.

	Subtheme	Student	Faculty
Instrumental Support	Access to Resources*	“As far as supporting me, whenever I do need anything, like a letter of rec or just some time to talk to him, I just have to let him know, because he’s super busy…. So in that sense, he’s very supportive, and always sends us opportunities to the undergrads, if there’s any, through email.” – Karmen	*Not emphasized by faculty*
	Research Collaboration*	“And I think he puts a lot of responsibility in us to figure things out on our own or contribute towards the research on our own. In the weekly meetings, we would be given tasks to figure things out on our own... I personally was working with a graduate student towards a new part of our research where we were trying to start everything up from the ground up, trying to figure out how we can record various things in different ways. And I think I was definitely a key part of the process of figuring out how we can get stuff to work. So I think in that way he definitely does care about the way we think.” – Lucas	“I think a lot of the independent research and senior thesis type projects do come from a question that an undergraduate asked in one of these meetings about something that either a graduate student or a different undergraduate was working on. And then, “Oh, you seemed interested in this. Do you want to follow up on that and actually do an independent project?” – Lexi
Psychosocial Support	Community**	*Not emphasized by students*	“Well, we try to always to do some building activities, like team building activities. I don’t know. For instance, last lab meeting, my grad students wanted to do with all the lab, like talking about our childhood. Everybody sent a picture of when they were kids to one of the grad students, and we needed to guess who was in all the pictures. It was really fun. Everybody was joking. That gave the opportunity for people to share.” – Nancy
	Rapport**	“I think they care about how I feel, because my faculty mentor will ask me to get food, so they’ll take me out, and they’ll ask me about classes, what classes I should take next year.” – Michael	“I do take a personal interest in my students, and I always remind them they should share with whatever they feel comfortable. But we do talk about summer plans, how their families are doing, how they’re doing in terms of their physical health, their mental health. To the extent they feel comfortable, they share with me if they get engaged, if their dog has a baby... I check up on them regularly.” – Amelia
	Barriers*	“If I would want more of an interaction, I would have to schedule time to let him know that that I want to talk about something. Or specifically a one-on-one conversation, it would be more so me reaching out. And then, yeah, the only other time I talk to him is during our lab meetings. But I’m more so like an auditor, sitting in, just kind of listening, as opposed to asking the questions.” – Karmen	*Not emphasized by faculty*

* Emphasized more by students.

** Emphasized more by faculty.

**Access to resources.** Students (n = 5) discussed how their mentors shared and encouraged them to apply to different professional opportunities, including jobs, scholarships, and conference presentations. Students also appreciated their mentors’ willingness to review and edit their resumes, personal statements, applications materials for graduate school, and conference presentations.

“She is always actively looking for opportunities for me to participate in things like different presentations, or she’s the one that introduced me to CAMP. So she’s just always looking to give me different opportunities and helping me make edits on my posters, or helping me make edits on my slides, or it doesn’t even have to be involved in research. She just helps me with other applications in general.” – Eric

**Research collaboration.** Students (n = 4) felt supported and valued by their mentors in their research projects. Students demonstrated a sense of ownership and agency when discussing responsibilities and contributions to their work. Brittany described her experiences of formulating a hypothesis and designing an experiment with the help of her mentor.

“He is not telling me what to do. He’s just asking me questions. Every time we meet, I’ve come a long way. He’s told me this. Between the first meeting where I had all these ideas and the research actually getting to the point where I’m now doing research... He’s never told me, ‘This is what you need to be thinking about.’ He’s just asking questions about how... ‘Well, what about this? Or what about that?’ And I have done everything. I have formulated my own hypothesis. I formulated my own methodology. I’m doing all the research. It’s kind of like the guidance.” – Brittany

Similarly, Lucas described his mentor’s expectations and how he contributed significantly to their research project. His mentor showed him caring by trusting him to figure it out on his own. Lucas felt validated in his critical thinking skills.

“And I think he puts a lot of responsibility in us to figure things out on our own or contribute towards the research on our own. In the weekly meetings, we would be given tasks to figure things out on our own... I personally was working with a graduate student towards a new part of our research where we were trying to start everything up from the ground up, trying to figure out how we can record various things in different ways. And I think I was definitely a key part of the process of figuring out how we can get stuff to work. So I think in that way he definitely does care about the way we think.” – Lucas

#### Psychosocial support.

Two students emphasized and provided a number of examples of psychosocial support, which focused on their socioemotional health, from their mentors. Other students (n = 3) included one example of their mentor’s psychosocial support. Students characterized psychosocial support from their mentors as rapport, addressing diversity, affirmation, and flexibility.

**Rapport.** A few students discussed how their faculty mentor tried to get to know them through informal conversations about their classes or personal lives. In doing so, mentors have established a more personal relationship with their students.

“I think they care about how I feel, because my faculty mentor will ask me to get food, so they’ll take me out, and they’ll ask me about classes, what classes I should take next year... I just feel like it’s a very natural and more like a father figure, like a family dynamic rather than an instructor dynamic.” – Michael

**Addressing diversity.** Two students appreciated their faculty mentor’s efforts and recognition that their needs may be different from other students based on their backgrounds.

“We come from different backgrounds. He definitely tries to understand. He asks about me, my family. I think he tries to understand me, but I think understanding someone is difficult unless you’ve had a similar background to them, but I will say, he tries to understand. He treats me the same as other graduate students, and he also understands that I may not have the same scientific background, and I may not have the same opportunities in high school and stuff like that. So, I think he understands at the basic level that I’m not the same.” – Michael

**Affirmation.** Two students described instances of when their mentors provided positive feedback when they were questioning their future career plans. The verbal affirmations from their mentor were important in building their confidence.

“Because I had a lot of setbacks with funding and I failed organic chemistry and that dropped my GPA to below the minimum level for applying for the Goldwater Research Scholarship, which is a really important and prestigious award. He told me, ‘Don’t worry about that. You’ll get up. I have a lot of confidence that you will have a good future, so don’t worry about the setbacks.’ He often times reassures me that I’m on the right track and that I’m doing a good work and that I have a future.” – Brittany

**Flexibility.** Two students recognized their mentor’s flexibility and understanding of the students’ priorities. They appreciated that their mentors recognized that their classes and health should be prioritized over research responsibilities.

“And then anytime I’ve been stressed out, he’s always said your health is number one. Or let’s say if I had an update for lab or something due, but it was really stressing me out because it was midterms week. First of all, here’s some really last minute questions. Second of all, I’m still working on this. I’m going to do it tomorrow, but I don’t know how good it’s going to be because I’m just swamped, and then he’ll just be like, sleep is number one. Don’t stay up to do this. It’s not pertinent, your health is more important.” – Sophia

#### Barriers.

Some students (n = 4) described negative interactions and experiences with their mentors, which led them to feel more disconnected from their mentors. Karmen and Justin had minimal interactions with their faculty mentors during their research experience. Karmen discussed how she was expected to initiate an interaction, if necessary, and that she was not an active participant during lab meetings.

“If I would want more of an interaction, I would have to schedule time to let him know that that I want to talk about something. Or specifically a one-on-one conversation, it would be more so me reaching out. And then, yeah, the only other time I talk to him is during our lab meetings. But I’m more so like an auditor, sitting in, just kind of listening, as opposed to asking the questions.” – Karmen

Justin described how his mentor was typically unavailable, even to offer congratulations when Justin had received an award. Justin perceived that he was not a priority for his mentor, and was unable to describe any positive experiences or support from his mentor.

I would say, as far as one-on-one interactions with my faculty mentor, there weren’t many of them, and most of the interactions actually happened through the graduate research group. So more times than not, they were unavailable. And there was even an occasion where I actually got an award… for the work that I was doing in this program. And the faculty mentor tried to arrange to meet with me and then couldn’t make one meeting, tried to reschedule for a second meeting, and then I ended up never actually having that face to face. So unfortunately I can’t speak as much on that.” – Justin

Lucas was confused by the lab hierarchy. His mentor allowed his graduate students to refer to him by his first name and seemingly had a more personal relationship with them. The relationship with undergraduate students was more professional and they had to refer to their mentor by their title, which created a barrier between their mentor.

“I think that at least my mentor, he became less professional with the graduate students. But when it came to research assistants, he had a very strict professional relationship with. For example, we had to call him Professor (First Name) or Professor (Last Name), and the graduate students could just call him (First Name). And I asked him about this as well because I was a bit confused. It’s kind of awkward sometimes when I’m saying “Professor (Last Name)” and someone else is saying “(First Name)” in the same room. And he elaborated with exactly what I said: graduate students can call him (First Name); research assistants and undergraduates, they keep it professional.” – Lucas

Rosa characterized conversations with her mentor as challenging and difficult to comprehend. Her mentor did not explain scientific jargon and failed to consider her level of background knowledge. Because her mentor did not make the research topic accessible, Rosa felt disconnected from her mentor.

“Okay. Because in his brain, he understands everything that he’s doing, and so he’ll just talk, talk, talk, and there’s a lot of scientific jargon, I would say. And I will try and follow along for a lot of the time, but it’s also really hard. And I feel like that that’s where our disconnect is, that I think he believes that I understand something really well, and then I have to keep asking questions or have him come back and explain it one more time and stuff like that.” – Rosa

**Different way of caring.** Brittany described meeting her mentor for the first time and how he asked her a lot of questions about her future plans. She felt unprepared for his way of caring and characterized her mentor’s style as “tough love”. It took her time to realize and adjust to his mentoring style.

“Well, he asked a lot of probing questions about what my plans were for the research and stuff. I didn’t know him very well, so at that moment I felt very vulnerable and I cried after the first meeting that we had, but then I realized that he was just trying to make me think about the research... He’s been treating me, not like an undergrad researcher. He’s treating me like a graduate researcher. He wants for me to think about things that if I am in front of a committee, how I would respond to that and how I would know that kind of stuff. I call it tough love, but he does it because he cares. I know he cares because he has told me that he has a lot of faith in me and that he thinks that I have a brilliant future. I know that he cares. It’s just that some people are more nurturing and some people are more tough love kind of love. If that makes sense.” – Brittany

### Faculty mentors’ perceptions of benevolence

To better understand the perception of their benevolence to students, we interviewed 10 faculty mentors and asked them to describe how they showed that they care about their students. Faculty mentors described providing psychosocial and instrumental support to their students.

#### Psychosocial support.

Almost all faculty mentors (n = 9) emphasized providing their students with psychosocial support. Mentors demonstrated this in one of two main methods: community and rapport.

**Community.** Seven of the 10 faculty mentors mentioned building community within their lab members. This was a priority for many faculty to ensure that their students felt valued and supported in their research labs. Lexi discussed her goal of creating a lab culture where all students contribute.

“I’m trying to create a culture where everyone thinks that they can be a critical part of the community and that you, everyone has different starting points, but that they still have something important to contribute and that people’s experiences are valid and important and that they… we value all kinds of contributions.” – Lexi

Faculty mentors built community in a variety of ways, including team-building exercises, sharing strengths during lab meetings, and organizing or hosting social events. Nancy included a team-building activity proposed by her graduate students. The activity created a light and fun environment in lab meeting for everyone to engage in and contribute to a non-science based discussion.

“Well, we try to always to do some building activities, like team building activities. I don’t know. For instance, last lab meeting, my grad students wanted to do with all the lab, like talking about our childhood. Everybody sent a picture of when they were kids to one of the grad students, and we needed to guess who was in all the pictures. It was really fun. Everybody was joking. That gave the opportunity for people to share.” – Nancy

Amelia described her efforts in building community through identifying individual strengths. With this asset-minded approach, she challenged undergraduate students to recognize their value in the research lab.

“Once a semester we also do have a circle where we sit down and talk about everyone’s strengths. I set the rules that no one can share their shortcomings, no one can share what they sucked at that semester. They have to share what is at least one strength that you see that you’re bringing to the team, and sometimes people emphasize their critical thinking skills or creative thinking skills. If they don’t, I do give them specific examples, like I think you are a very creative thinker, remember you came up with this idea that we incorporated into our experimental design. And it is what I try to encourage the most is independent thinking, which is a little bit hard with the undergraduates.” – Amelia

Three faculty mentors discussed organizing happy hours and other social events to celebrate graduations or publications. Bryce hosted lab meetings at his home during the summer to create a family environment for his students.

“One of the big things that I do with my laboratory is we have lab get togethers at my house. I bring them in. They know my family. In the summertime, we’ll have lab meetings on the back porch of the house, in front of the pool. Everybody comes in, they have more of a social get together as well… They’re the key component to the family environment part of it. That everybody who comes in is part of the family. It’s a different type of family because it’s a family you choose to join but just like your family who’s going to be there for you no matter what, that’s where we’ll get to as well. Especially if you make an equal commitment to us, we will be there for as long as you need us.” – Bryce

**Rapport.** Similar to the students’ perceptions, faculty mentors (n = 6) felt they spent significant time getting to know their students and building a personal relationship. Faculty mentors asked their students about classes, summer plans, mental health, and families. They recognized students would share as much as they would feel comfortable sharing.

“I try hard to get to know my students. As a mentor, I’m not sure that we know our students necessarily as well as other people in their lives, and I think that’s okay if they want to keep some things more private. But I do try to make sure that I’m open, and available, and willing to talk about things that students might want to bring up to me. I mean, I’m aware of when students in the lab are having financial trouble or trouble at home, because again, we normalize that in lab meeting and talk about it. And then some people might not feel comfortable talking about that in lab meeting, but I think that opens the door to those types of conversations that’s a little bit easier to broach in one-on-one meetings.” – Megan

A couple of faculty mentors mentioned informal and formal interactions to check-in with their students. Informal interactions took place in the hallway, labs, or coffee hours. Formal interactions were scheduled one-on-one meetings with students.

“With my team, I am always asking them how they’re doing. They sometimes pass in front of my door because they do have a mentor that is a grad student. But, I mean, I always stop them and they always stop my office and they say hi, I say hi. I do have a one-on-one meeting with the undergrads, and specifically this CAMP student, at least twice a quarter when they are in a school quarter. When they come in the summer, then I meet them more often.” – Nancy

**Addressing diversity.** A few of the faculty mentors (n = 3) acknowledged how their own backgrounds were different from their students. The mentors discussed being open to learning about and empathizing with their students. They recognized the importance of supporting minoritized students to create a more inclusive STEM community.

“I’d like to understand them. I think it’s hard to actually say that I fully do. I think I try to at least present an openness to understanding. I don’t think that it would be fair to say that without having the lived experiences of all of my students, that I can fully understand what they’re going through. I’m a multi-generation, my parents and my grandparents, my great-grandparents went to college and I come from a background that’s very privileged. And so I think it’s important to work with programs like CAMP, because I want people to all fell like they can be a part of science and see science as an inclusive community.” – Lexi

#### Barriers.

While most emphasized getting to know their students on a personal level, two faculty mentors discussed the importance of maintaining a professional relationship. Kaitlin described the challenges of being a woman in science and how she was treated differently by her colleagues. This played a significant role in her approach to engaging with her students and maintaining a professional boundary. She was cautious in building personal relationships with her students.

“I would say that interestingly, as a woman in science, I have my own kind of struggles. And so, especially when I first started as an assistant professor, it was hard for me because everyone thought I looked like a student and no one thought I was the professor. And so I feel like for better or worse, I often worked to not take too much of a personal interest in the sense that I wanted to be seen as a mentor and not as just one of the friends. And especially because of my colleagues not perceiving me as a professor at times, I feel like it was important for me to try to take a personal interest, but not overly so. So I’ve kind of reflected and try to find that line where I know enough about the students.” – Kaitlin

Kyle explicitly stated he was not trying to build a personal relationship with his students and did not take an interest in their personal lives.

“Well, I don’t really take a personal interest from the perspective that I’m not really building a relationship with the CAMP students. I want them to succeed. I want them to benefit from it. But I usually don’t ask a whole lot of questions about their personal lives.” – Kyle

#### Instrumental support.

More than half the faculty mentors (n = 6) described providing their students with instrumental support. Mentors discussed doing this in one of two main methods: goal setting and career planning and research collaboration.

**Goal setting and career planning.** Faculty mentors (n = 3) emphasized helping their students with identifying and supporting their career or research goals. Kyle described his method of caring about students was by asking about and ensuring they met their research goals.

“I show them that I care, well, by leading with their goals. I make it clear that the undergraduate research experience is really about them and them reaching their goals, not about productivity, not about impressing anyone. It’s really about helping them identify what they want to do, develop some skills, and then possibly develop some deliverables that can help them increase their options down the line. So I try to make a point to lead with asking them what they think when we talk about things, try to center, give them a chance to express themselves.” – Kyle

**Research collaboration.** Similar to students’ perceptions, some of the faculty mentors (n = 3) discussed being invested in developing their students’ research skills. Mentors valued their students’ critical thinking skills and contributions to their research projects.

“A lot of what I do is kind of let students develop their own research directions, their own questions asked, even if they’re not very good ones, it’s still an important process to learn how to think for yourself, how to kind of go through that. Then when you get the results that weren’t quite what you expected, okay, well what do we learn from this? What’s the next step? I think in general, just kind of forcing that or kind of enforcing the, “I’m just an advisor, I’m just a mentor. I’m just a sounding board. You’re the one doing the work. What direction do you think we should go in?”, if you are very, very consistent about that, pro or con, I think hopefully that gets the message across.” – Joel

## Discussion

In this study, we surveyed how STEM faculty mentors and minoritized students perceived the mentor’s ability and benevolence in their relationships. Students rated the faculty mentors’ abilities higher than the faculty mentors themselves, suggesting they trust their mentors’ abilities to do research. This is consistent with undergraduate perceptions of faculty as being confident, knowledgeable, and experts in their field [43,44]. In contrast, students rated the faculty mentors’ benevolence lower compared to the faculty mentors, suggesting students are more uncertain of their mentors’ benevolence.

One possible factor in the higher rating of benevolence by faculty mentors is the effect of social desirability bias, where responses are provided to show the respondent more favorably and particularly prevalent in sensitive topics [45]. While responding to survey items focused on caring about their students, faculty mentors may have rated themselves higher due to social desirability. Social desirability bias needs to be considered as a major factor in evaluating beliefs about inclusive education, and the extent of the bias depends on context [46]. For example, the respondent’s perception of the organization administering the survey is associated with how the respondent views inclusive education [46]. In this study, the survey was administered by the CAMP organization, which may have contributed to the social desirability bias in faculty mentors’ survey responses about benevolence.

To better understand their survey responses, follow-up interviews were used to not only assess whether faculty mentors cared about their students, but also how they communicated benevolence to their students. The interviews provided additional evidence for a difference in how benevolence was communicated in the relationship. In interviews, faculty mentors were more likely to emphasize psychosocial support compared to instrumental support. Conversely, students were more likely to emphasize instrumental support compared to psychosocial support provided by their faculty mentors. Instrumental support was strongly associated with faculty mentor’s ability. URE helps students build their human and cultural capital by developing their technical and research skills [44], which may prime students to more readily recognize instrumental support from faculty mentors. Faculty identify patience, honesty, empathy, and communication as extremely important qualities as a mentor; in contrast, feeling connected to their mentees is rated as moderately important [47].

Students’ emphasis on instrumental support from their mentors in the interviews may also partially be attributed to their mentors’ gender. Due to self-selection, most students interviewed described support from a male faculty mentor; most faculty mentors interviewed were women. Prior studies show that students’ perceptions of support differ from male and female mentors with mentees describing instrumental support more with male mentors than female mentors [48]. Similarly, a mentor’s approach and behavior towards mentees may depend on the gender of the mentor [49]. Male and White faculty are less likely to value URE for students compared to female and minoritized faculty [50]. Male faculty mentors are also less likely to convey empathy towards their mentees compared to female faculty [51]. Female mentors may also be particularly attentive to providing psychosocial support, given their own experiences of bias and feeling unrecognized in STEM fields [52].

A faculty mentor’s psychosocial support for students depends on a variety of factors [18]. Depending on the gender and race/ethnicity, mentees receive varying levels of psychosocial support from their mentors [53]. Female mentees are more likely to receive psychosocial support compared to male mentees. Asian mentees are less likely to receive psychosocial support compared to White mentees. Similarly, in the classroom, women of color are less likely to report their faculty care about them compared to White women [54].

Given the different identities of students, effective mentoring requires faculty mentors to be intentional and specific to students [18]. Faculty mentors described providing psychosocial support to their students by building community, creating rapport, and addressing diversity. Faculty mentors spoke broadly about supporting their students and it was unclear how their approach was personalized to the needs of their minoritized undergraduate student. With respect to diversity, faculty mentors acknowledged differences between them and their students, but they did not explain how they helped their students navigate the complicated environment of academia. This is consistent with the experiences of minoritized graduate students, who describe never having discussions on race-related issues with their faculty mentors [55]. Faculty mentors need to learn how to have conversations about difficult topics to better support their minoritized students.

Building trust with students to have these conversations requires time, which is a limited resource for faculty. Time was most likely the reason why a couple of students had minimal interactions with their faculty mentors. Different mentoring structures exist in research labs, and some require little to no interaction with faculty mentors [17]. For example, with Justin, he felt his faculty mentor did not care about him because the mentor did not make time to meet with him. It is important to note that minoritized students are interested in engaging with faculty in a meaningful way, beyond a superficial relationship [30]. Students want more encouragement and psychosocial support from their faculty mentors [29]. Faculty mentors may consider discussing mentoring needs, expectations, and styles early on with their students to establish an effective mentoring relationship [18].

### Limitations

A major challenge with investigating a relationship from both student and faculty mentor perspectives was the availability of both parties. Due to self-selection, students and faculty mentors who participated in the interviews were not matched. For instance, students described their relationship with their faculty mentors, who did not choose to participate in the interview. Conversely, faculty mentors were interviewed but their students were not interviewed. It is possible that if we had been able to interview both students and their faculty mentors in a given relationship, then their ratings and descriptions of the faculty mentors’ benevolence may have been more similar. Some of the disconnect in communicating benevolence between faculty mentor and student descriptions may be attributed to the self-selection process of the interviews.

This study provided a limited sampling of student and faculty interview participants at research-intensive universities. It is important to consider the self-selection bias and how faculty mentors who participated in the interviews were most likely ones who are invested in mentoring students as they took time to participate in the interview. Faculty use a variety of mentorship structures and approaches [18] and future studies are needed to determine how faculty and students perceive benevolence in different mentoring relationships. Furthermore, the institutional context contributes to the nature of mentoring configurations [18]. For example, at research-intensive universities, students are likely interacting with graduate student or postdoctoral mentors in addition to their faculty research mentor. The perspectives and relationships between students and faculty mentors may be different based on institution type (i.e., research-intensive, primarily undergraduate institution, etc.). Future studies may investigate whether findings in this study are broadly applicable across institution types.

This study focused on the experiences of students in the CAMP program, which is designed to support minoritized students. In contrast, faculty research mentors primarily identified as White or Asian. Given the difference in race/ethnicity between students and faculty, it is possible that the perceptions of benevolence may be due to cultural differences. Prior studies have shown that faculty mentors’ views of culture vary and may contribute to a student’s experience in the lab or mentoring relationship [56,57]. It is also unknown whether age or generational differences contributes to how students and faculty perceive benevolence. Further studies are needed to determine how factors including mentor’s background and training, cultural backgrounds, and other demographic characteristics may contribute to benevolence in a mentoring relationship.

## Conclusion

Mentoring relationships have a significantly positive impact on outcomes for minoritized undergraduate students. Trust in the relationship is based on both ability and benevolence. The benevolence aspect of the mentoring relationship is potentially overlooked by faculty mentors in STEM. This study highlights the need for faculty mentors to consider how they communicate caring and psychosocial support to their students.

## Supporting information

S1 File This file contains survey items for students and faculty. (DOCX)

S1 Table Pseudonym and demographic information for interview participants. (DOCX)

S2 Table Interview analysis codebook. (DOCX)
