# Supplementary material for: Minoritized students and their faculty research mentors view benevolence differently in the relationship
Source: PLoS One. 2025 Sep 9;20(9):e0332153. doi: 10.1371/journal.pone.0332153 (PMC12419617; doi:10.1371/journal.pone.0332153)
Supplement: S1 File — (DOCX) [file pone.0332153.s001.docx]

Supplemental Information

**Student Survey Items**

Ability Items:

- This person is a knowledgeable teacher.
- This person is widely respected for their scholarly expertise.
- This person is a good mentor.

Benevolence Items:

- This person cares about how I feel.
- This person takes a personal interest in me.
- This person values my thinking.

**Faculty Survey Items**

Ability Items:

- I am a knowledgeable teacher.
- I am widely respected for their scholarly expertise.
- I am a good mentor.

Benevolence Items:

- I care about how my student feels.
- I take a personal interest in my student.
- I value my student’s thinking.

Student_covitality_trust

Start of Block: Consent

Q1 This is a research study about understanding how and where students receive support, including from their college and research mentors. Dr. Richard Cardullo from the UCR Department of Evolution, Ecology, and Organismal Biology will conduct this study. Research studies include only those people who choose to participate in the study. 


You are being asked to take part in this study because you are participating in faculty mentored research. To participate in this study, you must be over 18 years of age and be able to read and respond in English.


**Purpose**
This is a research study to determine how and where students receive support, including from their college and research mentors.
  
**Procedures:**
Participants in this study will be asked to complete an online survey, which is expected to take 20 minutes to complete. We will keep your answers confidential and will not share your personal information with anyone outside the research team. If you decide to discontinue participation altogether, your data will be deleted. If you choose to withdraw from the study, you will be allowed to keep the $15 gift certificate, in the event that you win the drawing.

 **Risks:**
Risks of this study are minimal. Some of the foreseeable risks or discomforts may include being potentially triggered by a question. You may skip questions you do not want to answer or stop participating at any time.
  
**Benefits:**
 You will not directly benefit from this research. Some of the benefits that may be expected include being able to provide better support to future students involved in research.

 **Alternatives:**
 Your alternative to participating in this research study is to not participate.

 **Compensation:**
You will be entered into a drawing to win one of ten $15 Amazon gift certificate. Winners will be notified two weeks after the survey deadline.

You must complete the survey to be entered into the drawing. You may only enter the drawing one time.
 **Voluntary Participation:**
 Your participation in this study is voluntary. You can decide to participate or not to participate, or to withdraw from it at any point without penalty or loss of benefits to which you are otherwise entitled to or already have.


**Whom can I talk to?**
If you have questions, concerns, or complaints, or think the research has hurt you, talk to the research team at Dr. Richard Cardullo (951-827-5782). If you have questions about your rights or complaints as a research subject, please contact the IRB Chairperson at 951-827-4802 during business hours or via email at irb@ucr.edu.


**Consent**
Participation in research is voluntary. The decision to participate or not participate is solely up to you.
If you wish to participate in this study, please check the the appropriate box below.

- I consent to participating in this research study. (1)
- I do not consent to participating in this research study. (2)

Skip To: End of Survey If This is a research study about understanding how and where students receive support, including fr... = I do not consent to participating in this research study.

End of Block: Consent

Start of Block: Default Question Block

Q2 After reading each item, please choose the response that best describes you.

|  | Very much unlike me (1) | Unlike me (2) | Somewhat unlike me (3) | Somewhat like me (4) | Like me (5) | Very much like me (6) |
| --- | --- | --- | --- | --- | --- | --- |
| I feel for my friends who are afraid or nervous about graduating. (1) |  |  |  |  |  |  |
| I stay focused while studying despite distractions. (2) |  |  |  |  |  |  |
| I have a friend at my college who cares about me. (3) |  |  |  |  |  |  |

Q3 After reading each item, please choose the response that best describes you.

|  | Very much unlike me (1) | Unlike me (2) | Somewhat unlike me (3) | Somewhat like me (4) | Like me (5) | Very much like me (6) |
| --- | --- | --- | --- | --- | --- | --- |
| I am able to stay positive even when facing uncertain situations. (1) |  |  |  |  |  |  |
| I persist on tasks that I cannot immediately complete. (2) |  |  |  |  |  |  |
| I think before I act. (3) |  |  |  |  |  |  |

Q4 After reading each item, please choose the response that best describes you.

|  | Very much unlike me (1) | Unlike me (2) | Somewhat unlike me (3) | Somewhat like me (4) | Like me (5) | Very much like me (6) |
| --- | --- | --- | --- | --- | --- | --- |
| I appreciate those who are close to me. (1) |  |  |  |  |  |  |
| I can wait for what I want. (2) |  |  |  |  |  |  |
| I recognize my moods and feelings. (3) |  |  |  |  |  |  |

Q5 After reading each item, please choose the response that best describes you.

|  | Very much unlike me (1) | Unlike me (2) | Somewhat unlike me (3) | Somewhat like me (4) | Like me (5) | Very much like me (6) |
| --- | --- | --- | --- | --- | --- | --- |
| I think about potential consequences before I act. (1) |  |  |  |  |  |  |
| I am aware of others' hardships. (2) |  |  |  |  |  |  |
| I feel like there is a strong feeling of togetherness on my campus. (3) |  |  |  |  |  |  |

Q6 After reading each item, please choose the response that best describes you.

|  | Very much unlike me (1) | Unlike me (2) | Somewhat unlike me (3) | Somewhat like me (4) | Like me (5) | Very much like me (6) |
| --- | --- | --- | --- | --- | --- | --- |
| I have a good sense of why I have certain feelings most of the time. (1) |  |  |  |  |  |  |
| My family gets along well with each other. (2) |  |  |  |  |  |  |
| My family continues to love and support one another in tough situations. (3) |  |  |  |  |  |  |

Q7 After reading each item, please choose the response that best describes you.

|  | Very much unlike me (1) | Unlike me (2) | Somewhat unlike me (3) | Somewhat like me (4) | Like me (5) | Very much like me (6) |
| --- | --- | --- | --- | --- | --- | --- |
| I do not stop my work even if it is very difficult. (1) |  |  |  |  |  |  |
| My friends describe me as full of life. (2) |  |  |  |  |  |  |
| I am able to identify the motivations behind my actions. (3) |  |  |  |  |  |  |

Q8 After reading each item, please choose the response that best describes you.

|  | Very much unlike me (1) | Unlike me (2) | Somewhat unlike me (3) | Somewhat like me (4) | Like me (5) | Very much like me (6) |
| --- | --- | --- | --- | --- | --- | --- |
| Each day I look forward to having a lot of fun. (1) |  |  |  |  |  |  |
| I appreciate the relationships I have developed throughout my life. (2) |  |  |  |  |  |  |
| I am able to think about the alternatives to a problem under stressful situations. (3) |  |  |  |  |  |  |

Q9 After reading each item, please choose the response that best describes you.

|  | Very much unlike me (1) | Unlike me (2) | Somewhat unlike me (3) | Somewhat like me (4) | Like me (5) | Very much like me (6) |
| --- | --- | --- | --- | --- | --- | --- |
| When I feel down, I try to focus on the positives. (1) |  |  |  |  |  |  |
| I can talk to my friends about pretty much anything. (2) |  |  |  |  |  |  |
| I feel like I belong at this university. (3) |  |  |  |  |  |  |

Q10 After reading each item, please choose the response that best describes you.

|  | Very much unlike me (1) | Unlike me (2) | Somewhat unlike me (3) | Somewhat like me (4) | Like me (5) | Very much like me (6) |
| --- | --- | --- | --- | --- | --- | --- |
| I have a friend who gives me the emotional support I need. (1) |  |  |  |  |  |  |
| I approach life with excitement and energy. (2) |  |  |  |  |  |  |
| When I reflect on my life, there is much to be grateful for. (3) |  |  |  |  |  |  |

Q11 After reading each item, please choose the response that best describes you.

|  | Very much unlike me (1) | Unlike me (2) | Somewhat unlike me (3) | Somewhat like me (4) | Like me (5) | Very much like me (6) |
| --- | --- | --- | --- | --- | --- | --- |
| There is a sense of togetherness within my family. (1) |  |  |  |  |  |  |
| Generally, I feel capable of overcoming obstacles. (2) |  |  |  |  |  |  |
| I can lift my mood by redirecting my thoughts to positive ideas. (3) |  |  |  |  |  |  |

Q12 After reading each item, please choose the response that best describes you.

|  | Very much unlike me (1) | Unlike me (2) | Somewhat unlike me (3) | Somewhat like me (4) | Like me (5) | Very much like me (6) |
| --- | --- | --- | --- | --- | --- | --- |
| I will be able to achieve most of the goals that I have set for myself. (1) |  |  |  |  |  |  |
| I feel energetic in my life right now. (2) |  |  |  |  |  |  |
| I feel badly when my friends are put down. (3) |  |  |  |  |  |  |

Q13 After reading each item, please choose the response that best describes you.

|  | Very much unlike me (1) | Unlike me (2) | Somewhat unlike me (3) | Somewhat like me (4) | Like me (5) | Very much like me (6) |
| --- | --- | --- | --- | --- | --- | --- |
| Outside of my friends, there are other people on campus who care about my well-being. (1) |  |  |  |  |  |  |
| I will be able to successfully overcome many challenges. (2) |  |  |  |  |  |  |
| I usually expect to have a good day. (3) |  |  |  |  |  |  |

End of Block: Default Question Block

Start of Block: Block 1

Q14 Who is your faculty research mentor? Please provide the first and last name.

________________________________________________________________

Q15 When did you start working with your faculty research mentor? Please provide the month and year.

________________________________________________________________

Q16 In a typical month BEFORE covid, how often did you interact with your faculty research mentor?

- more than once a week (1)
- once a week (2)
- once every two weeks (3)
- once every three weeks (4)
- once a month (5)
- less than once a month (6)
- not applicable (7)

Q17 In a typical month BEFORE covid, how did you primarily interact with your faculty research mentor?

- Email (1)
- Phone (2)
- Zoom/online (3)
- In person (4)
- Not applicable (5)

Q18 In a typical month AFTER covid, how often did you interact with your faculty research mentor?

- more than once a week (1)
- once a week (2)
- once every two weeks (3)
- once every three weeks (4)
- once a month (5)
- less than once a month (6)
- not applicable (7)

Q19 In a typical month AFTER covid, how did you primarily interact with your faculty research mentor?

- Email (1)
- Phone (2)
- Zoom/online (3)
- In person (4)
- Not applicable (5)

Q20 We would like to know who has helped you in your research experience. From the list below, select **all** those whom you consult about your research.

- Faculty (1)
- Postdoctoral fellow (2)
- Graduate student (3)
- Undergraduate student (4)
- Other (please type below) (5) ________________________________________________

Q21 From the list above, who has been the MOST HELPFUL in your research experience? (select one)

- Faculty (1)
- Postdoctoral fellow (2)
- Graduate student (3)
- Undergraduate student (4)
- Other (please type below) (5) ________________________________________________

Q22 With your **faculty research mentor** in mind, please rate each of the following statements.

|  | Strongly disagree (1) | Somewhat disagree (2) | Neither agree nor disagree (3) | Somewhat agree (4) | Strongly agree (5) |
| --- | --- | --- | --- | --- | --- |
| This person is a knowledgeable teacher. (1) |  |  |  |  |  |
| This person is a good mentor. (2) |  |  |  |  |  |
| This person is widely respected for their scholarly expertise. (3) |  |  |  |  |  |
| I respect this person's skill at teaching. (4) |  |  |  |  |  |
| I respect this person as a mentor. (5) |  |  |  |  |  |
| This person cares about how I feel. (6) |  |  |  |  |  |
| This person understands me. (7) |  |  |  |  |  |

Q23 With your **faculty research mentor** in mind, please rate each of the following statements.

|  | Strongly disagree (1) | Somewhat disagree (2) | Neither agree nor disagree (3) | Somewhat agree (4) | Strongly agree (5) |
| --- | --- | --- | --- | --- | --- |
| This person respects me. (1) |  |  |  |  |  |
| This person takes a personal interest in me. (2) |  |  |  |  |  |
| This person values my thinking. (3) |  |  |  |  |  |
| This person tries to be fair in dealings with me. (4) |  |  |  |  |  |
| I can count on this person to live up to his/her/their word. (5) |  |  |  |  |  |
| This person is honest with me. (6) |  |  |  |  |  |
| This person tries to do the right thing for me. (7) |  |  |  |  |  |

End of Block: Block 1

Start of Block: Block 2

Q24 We would like to know about your perceptions of the campus climate. Think about your perceptions of the overall climate in the college. Consider your personal interactions and experiences with the college faculty, instructors, and teaching assistants this past academic year. 
Given your experiences, please rate the following statements.

|  | Strongly disagree (1) | Somewhat disagree (2) | Neither agree nor disagree (3) | Somewhat agree (4) | Strongly agree (5) |
| --- | --- | --- | --- | --- | --- |
| The college is widely respected for its scholarly expertise. (1) |  |  |  |  |  |
| The college values the importance of quality academic work. (2) |  |  |  |  |  |
| The college has the know-how to help students succeed. (3) |  |  |  |  |  |
| The college faculty are skilled teachers. (4) |  |  |  |  |  |
| The college faculty are good mentors. (5) |  |  |  |  |  |
| The college understands students like me. (6) |  |  |  |  |  |
| The college cares about students like me. (7) |  |  |  |  |  |

Q25 We would like to know about your perceptions of the campus climate. Think about your perceptions of the overall climate in the college. Consider your personal interactions and experiences with the college faculty, instructors, and teaching assistants this past academic year. 
Given your experiences, please rate the following statements.

|  | Strongly disagree (1) | Somewhat disagree (2) | Neither agree nor disagree (3) | Somewhat agree (4) | Strongly agree (5) |
| --- | --- | --- | --- | --- | --- |
| The college respects students like me. (1) |  |  |  |  |  |
| The college looks out for the well-being of students like me. (2) |  |  |  |  |  |
| The college helps students like me succeed. (3) |  |  |  |  |  |
| The college does the right thing for students like me. (4) |  |  |  |  |  |
| The college tries to be fair in its dealings with students like me. (5) |  |  |  |  |  |
| When the college says they're going to do something, they do it. (6) |  |  |  |  |  |
| The college is watching out for my best interests. (7) |  |  |  |  |  |
| The college is honest with me. (8) |  |  |  |  |  |

End of Block: Block 2

Start of Block: Block 3

Q26 Please rate the following statements.

|  | Strongly disagree (1) | Somewhat disagree (2) | Neither agree nor disagree (3) | Somewhat agree (4) | Strongly agree (5) |
| --- | --- | --- | --- | --- | --- |
| College is boring but I get through it because I know I need a degree. (1) |  |  |  |  |  |
| I want people to know about my educational background. (2) |  |  |  |  |  |
| I want people to respect my educational background. (3) |  |  |  |  |  |
| I am genuinely interested in the material I study in college. (4) |  |  |  |  |  |
| I do what I need to for the grade without caring about understanding the material. (5) |  |  |  |  |  |
| I go to college primarily because I need a degree to get a good job. (6) |  |  |  |  |  |
| Learning is one of my passions. (7) |  |  |  |  |  |

End of Block: Block 3

Start of Block: Block 4

Q27 What is your major?

________________________________________________________________

Q28 What is your gender?

- Female (1)
- Male (2)
- Non-binary (3)
- Other (please type below) (4) ________________________________________________
- Decline to state (5)

Q29 What is your ethnicity? (select all that apply).

- American Indian/Alaskan Native (1)
- Asian (2)
- Black/African American (3)
- Chicano/Latinx (4)
- Native Hawaiian/Pacific islander (5)
- White (6)
- Other (please type below) (7) ________________________________________________
- Decline to state (8)

Q30 Are you a first-generation student?

- Yes (1)
- No (2)
- Decline to state (3)

Q31 Are you a transfer student

- Yes (1)
- No (2)
- Decline to state (3)

Q32 Are you affiliated with a research program? Select all that apply.

- CAMP (1)
- MARCUSTAR (2)
- McNair (3)
- RISE (4)
- UC LEADS (5)
- Other (6) ________________________________________________

Q33 Name

________________________________________________________________

Q34 What is your student identification number?

________________________________________________________________

Q35 What is your email address? This will be used to contact you if you win one of the gift certificates.

________________________________________________________________

End of Block: Block 4
