# Supplementary material for: Minoritized students and their faculty research mentors view benevolence differently in the relationship
Source: PLoS One. 2025 Sep 9;20(9):e0332153. doi: 10.1371/journal.pone.0332153 (PMC12419617; doi:10.1371/journal.pone.0332153)
Supplement: S1 Table — (DOCX) [file pone.0332153.s002.docx]

**S1 Table. Pseudonym and demographic information for interview participants**

| Pseudonym | Student/Faculty | Gender | Self-reported ethnicity |
| --- | --- | --- | --- |
| Ana | Student | Female | Black/African American and Middle Eastern |
| Brittany | Student | Female | Chicano/Latinx |
| Daniel | Student | Male | Chicano/Latinx |
| Eric | Student | Male | Chicano/Latinx and White |
| Justin | Student | Male | Black/African American and Native Hawaiian/Pacific Islander |
| Karmen | Student | Female | Chicano/Latinx |
| Lucas | Student | Male | Chicano/Latinx |
| Michael | Student | Female | Black/African American |
| Noah | Student | Male | Chicano/Latinx |
| Rosa | Student | Female | Chicano/Latinx |
| Sophia | Student | Female | American Indian/Alaskan Native, Asian, and White |
| Amelia | Faculty | Female | White |
| Bryce | Faculty | Male | White |
| Joel | Faculty | Male | White |
| Kaitlin | Faculty | Female | White |
| Kyle | Faculty | Male | White |
| Lauren | Faculty | Female | White |
| Lexi | Faculty | Female | White |
| Megan | Faculty | Female | White |
| Nancy | Faculty | Female | Chicano/Latinx |
| Penelope | Faculty | Female | White |
