# Supplementary material for: Minoritized students and their faculty research mentors view benevolence differently in the relationship
Source: PLoS One. 2025 Sep 9;20(9):e0332153. doi: 10.1371/journal.pone.0332153 (PMC12419617; doi:10.1371/journal.pone.0332153)
Supplement: S2 Table — (DOCX) [file pone.0332153.s003.docx]

**S2 Table. Interview analysis codebook**

| Theme | Code | Description of code | Example quotes |
| --- | --- | --- | --- |
| Instrumental Support | Access to Resources | Faculty mentors provides and shares resources with students. | “She is always actively looking for opportunities for me to participate in things like different presentations, or she's the one that introduced me to CAMP. So she's just always looking to give me different opportunities and helping me make edits on my posters, or helping me make edits on my slides, or it doesn't even have to be involved in research. She just helps me with other applications in general.” – Eric (student) |
|  | Research Collaboration | Faculty mentor and students recognize student’s efforts and contributions to the research project. | “He is not telling me what to do. He's just asking me questions. Every time we meet, I've come a long way. He's told me this. Between the first meeting where I had all these ideas and the research actually getting to the point where I'm now doing research... He's never told me, "This is what you need to be thinking about." He's just asking questions about how ... "Well, what about this? Or what about that?" And I have done everything. I have formulated my own hypothesis. I formulated my own methodology. I'm doing all the research. It's kind of like the guidance.” – Brittany (student) |
|  | Goal Setting & Career Planning | Faculty mentor supports and advises students on their career plans and goals. | “I show them that I care, well, by leading with their goals. I make it clear that the undergraduate research experience is really about them and them reaching their goals, not about productivity, not about impressing anyone. It's really about helping them identify what they want to do, develop some skills, and then possibly develop some deliverables that can help them increase their options down the line. So I try to make a point to lead with asking them what they think when we talk about things, try to center, give them a chance to express themselves.” – Kyle (faculty) |
| Psychosocial Support | Community | Faculty mentor fosters an environment for members of the lab to build connections with each other and ensures everyone has opportunities to contribute. | “Once a semester we also do have a circle where we sit down and talk about everyone's strengths. I set the rules that no one can share their shortcomings, no one can share what they sucked at that semester. They have to share what is at least one strength that you see that you're bringing to the team, and sometimes people emphasize their critical thinking skills or creative thinking skills. If they don't, I do give them specific examples, like I think you are a very creative thinker, remember you came up with this idea that we incorporated into our experimental design. And it is what I try to encourage the most is independent thinking, which is a little bit hard with the undergraduates.” – Amelia (faculty) |
|  | Rapport | Faculty mentor and students build a personal relationship. | “I try hard to get to know my students. As a mentor, I'm not sure that we know our students necessarily as well as other people in their lives, and I think that's okay if they want to keep some things more private. But I do try to make sure that I'm open, and available, and willing to talk about things that students might want to bring up to me. I mean, I'm aware of when students in the lab are having financial trouble or trouble at home, because again, we normalize that in lab meeting and talk about it. And then some people might not feel comfortable talking about that in lab meeting, but I think that opens the door to those types of conversations that's a little bit easier to broach in one-on-one meetings.” – Megan (faculty) |
|  | Addressing Diversity | Faculty mentor acknowledges diverse backgrounds (i.e, race/ethnicity, socioeconomic status, etc.) of students. | “I’d like to understand them. I think it’s hard to actually say that I fully do. I think I try to at least present an openness to understanding. I don’t think that it would be fair to say that without having the lived experiences of all of my students, that I can fully understand what they’re going through. I’m a multi-generation, my parents and my grandparents, my great-grandparents went to college and I come from a background that’s very privileged. And so I think it’s important to work with programs like CAMP, because I want people to all fell like they can be a part of science and see science as an inclusive community.” – Lexi (faculty) |
|  | Flexibility | Faculty mentor is understanding of student’s priorities. | “And then anytime I've been stressed out, he's always said your health is number one. Or let's say if I had an update for lab or something due, but it was really stressing me out because it was midterms week. First of all, here's some really last minute questions. Second of all, I'm still working on this. I'm going to do it tomorrow, but I don't know how good it's going to be because I'm just swamped, and then he'll just be like, sleep is number one. Don't stay up to do this. It's not pertinent, your health is more important.” – Sophia (student) |
| Barriers | Barriers | Faculty mentor and students have minimal interactions or lack a personal relationship. | “I would say, as far as one-on-one interactions with my faculty mentor, there weren't many of them, and most of the interactions actually happened through the graduate research group. So more times than not, they were unavailable. And there was even an occasion where I actually got an award from Thermo Fisher Scientific for the work that I was doing in this program. And the faculty mentor tried to arrange to meet with me and then couldn't make one meeting, tried to reschedule for a second meeting, and then I ended up never actually having that face to face. So unfortunately I can't speak as much on that.” – Justin (student) |
